# Directions for Using the Plastic Metallic Filling

**Published:** 1864-01

**Authors:** B. Wood

**Affiliations:** Albany, N. Y.


DENTAL REGISTER OF THE WEST.

Vol. XVIII.] JANUARY, 1864. [No. 1.

### Original Essays and Communications

### DIRECTIONS FOR USING THE PLASTIC METALLIC FILLING.

By B. Wood, M. D.

As the directions for using this material are not sufficiently full and explicit for some, and appear to be not well-understood by others, and the improved instruments employed modify the procedure to some extent, I have thought it might be of service, particularly to beginners, to submit a communication on the subject.* I shall endeavor to be as briefly to the point as possible.

The Plastic Metallic Filling is designed for making perfect and durable plugs—not for temporarily stopping up diseased and worthless teeth. Therefore let the cavity be well prepared, and the whole operation performed with the view of saving the tooth permanently.

Cut the metal with a plate sheers into little blocks, the same way as gold solder, scraping it in like manner to secure a clean surface,—the blocks to vary in size from one-twentieth to one-eighth of an inch. Lay these, separate from each other, on a paper card or other non-conducting support,

* Since this article was written for publication, a synopsis of it has been printed, and to some extent circulated among dentists using the material; but it may not be objectionable to have it in a more permanent and accessible form; while to many it will be the same as though the whole were now for the first communicated.

or, still better, on a warm slab of soapstone or porcelain; with your spirit-lamp within easy reach. Being provided with the proper instruments, select one to suit the cavity, and determine what motion to give it. Heat the bulb a few moments in the spirit flame, until the point or blade is rather hot to the touch—which by practice affords a delicate test. Now press the blade, or point, as the case may be, lightly upon a block of the metal—say one-third the size of the cavity—for a few seconds, until the blade, chilled by the contact, receives heat enough from the bulb to soften it, which will be indicated by its yielding to the pressure, when, on raising the instrument it will cling to it so that it may be carried in any position to the cavity. If it drops off, or, when the blade is tilted, rolls from side to side upon it, or melts down in globular shape, it is too fluid and must be allowed to cool a moment: learn to judge of this by the eye and touch in previous manipulation upon extracted teeth, etc. Test its plasticity and adjust it to the right spot on the instrument with the finger before your patient; it will quiet any apprehensions on his part, provided you exhibit none The proper condition is when it yields to light pressure, but otherwise scarcely changes its form. Then apply it securely to its place, and do not on any account hurry or slight the operation. When a portion has been introduced the rest may be used more fluid, adding it by sections. To favor adhesion, it is well to soften the surface of the plug with the reverse side of the plugger the moment of adding each piece.

The metal is to be applied in nearly the same manner as you would seal up a cavity with semifluid wax or plaster, and as though aiming to obtain a perfect cast of it,—securing first the bottom, walls, and undercut portions, wiping the instrument off against the margins. Having thus moulded it well in all around, build up from the center. In molar cavities, using a flat bladed instrument that will freely pass into them, you can, by making a circular sweep, secure one-half or two-thirds of the walls the first time; then taking up another block on the reverse side of the blade, secure the rest the same way: a few whirls with a cylindrical point, touching the circumference all round, effect the same end.

When the cavity is deep, fill first from the bottom until partly full,—carrying the metal upon the end of the cylindrical or square-pointed instrument; or put the blocks in with plugging forceps or tweezers, and mould them down with the heated instrument; but this requires some experience as to its proper temperature: then fill up as before described. If the metal congeals prematurely in the cavity, apply the heated instrument to it *in situ* and fuse it to its place; if this renders it too fluid, brush over quickly; when of the proper plasticity, work it in with gentle pressure where wanted. For finishing, a higher temperature is required,—sweeping the surface of the plug from the center toward the circumference. Should you subsequently discover an imperfection at the margin, soften the filling near the spot and press it home; then fill the indentation with a new piece, using it fluid enough to promote union. The greatest difficulty occurs near or under the edge of the gum; this is obviated by giving the instrument a peculiar sweep, upward or downward as the case may be, which will be acquired by practice; or take the first block of metal on the end of a round or square-pointed plugger and apply it to the distal extremity of the cavity, the same as to the bottom of deep crown-cavities. Be sure first to secure well this portion.

If hot water is used for heating the instruments, the bulbs must be large and the metal brought near or quite to fusion.*

---

* For, on removal from the heating medium, the instrument parts at once with a portion of caloric; and if applied at say 200° to the metal at say 60°, then, other things being equal, an equilibrium taking place between the two, the resultant temperature is 130°, or some 30° below fusion, [See, on these points, the Dental Register of the West, for May, 1863, page 203, in correction of a criticism in the American Dental Review, for January, copied in the Dental Cosmos of the same date; and which, as the correction was not copied in these journals, has served to mislead readers.]

Place the blocks on the cover of the vessel, or on a porcelain slab set over it, and heat the instruments dry in a separate apartment to prevent the need of wiping them. But this plan is inconvenient, and for large cavities requires the aid of the flame.

In applying the Filling, the cavity and metal should be kept dry. Place a bit of spunk or tissue-paper at the edge of the gum to absorb the exudation. Let your napkin cover the lips where working. The shafts of the instruments may be wrapped with gummed paper or a shred of dry cotton, etc.; but this precaution will be altogether unnecessary, after a little practice.

For the bottom of large deep cavities, near the nerve, asbestos may be applied rolled up in a bit of foil; or a layer of spunk, cork, etc., may be used previously dipped in creosote and dried; also, Hill's Stopping will prove useful; for, although it really requires a higher heat for working well than the metal, it transmits it less quickly, and when introduced affords a good non-conductor. If oxichloride of zinc or plaster be resorted to, seal up with wax until hard and dry.

A scraper is the best for trimming off the plug,—cutting from the center toward the margins: ordinary scalers answer very well, or can be readily ground to the proper shape: the file and bur will also prove useful in finishing. Then polish and burnish, the same as gold plugs.

Learn the use of the material by previous manipulation with it out of the mouth; practice these directions, and it will suggest others; get proficient in plain cases before undertaking the difficult; neglect no opportunity of practical instruction.

ALBANY, N. Y., Sept. 12, 1863.

To the foregoing I may add something in respect to the heat required in using the Filling—the only objection publicly urged; although what perhaps opposes as much, in practice, is that it involves a system of manipulation to which the profession are unaccustomed. Beginners often object to the difficulty of application; and in bad cases it certainly is no easy task. But this holds in the use of gold; and bear in mind the task to become proficient with gold, and also that earnest hands do succeed with the Filling after comparatively little practice, and that, too, in cases where a like success would not have been achieved with gold; while ordinary cases are soon managed with a degree of neatness and facility to afford the highest gratification both to the operator and his patient.

But whatever objection relates to the actual degree of heat required, is not to be overcome by skill and practice. In cases where it produces pain, it is so far objectionable, the same as excavating and other operations attended with pain, but to which we are accustomed. If it inflicted injury to the tooth, it would be a valid objection; but experience proves that it does not. But the *apprehension* excited by the idea of heat, whether in the mind of the operator or patient, so easily intensified by persons so disposed, presents a serious obstacle, which is only to be removed by familiarity. We are naturally distrustful of unusual means and procedures. Dentists, to whom the extraction of a tooth has become a small affair, will falter at the much less formidable operation of making an incision through the alveolus for the treatment of abscess, when a new thing to them. Many were seriously alarmed at the dangers of the mallet, who would throw two hundred pounds pressure upon a tooth without apprehension.

The actual heat required is not unusual, being less than that required by most substances used in a fluid or plastic condition. The metal softens and is fit for working between 150° and 160°. On cooling, it congeals at about 150°, not higher, according to any tests made, than 156°; and as the first piece introduced congeals on coming in contact with the tooth, and the subsequent pieces on coming in contact with that already in, it is evident that, when introduced by sections, the temperature of the tooth can not be raised higher than 150° to 156°, however large the cavity, or protracted the operation. This is borne, with little discomfort, in a dead tooth, but excites pain when the nerve is alive (for which reason it is proper to interpose a non-conductor). In ordinary cases, however, the heat being greatly lowered in diffusing itself throughout the entire tooth does not sensibly elevate its general temperature; and accordingly pain is not felt in such cases, except where the dentine is unusually irritable, and then it is but momentary and far less for the time than that produced by cutting away the decay.

